# The ability of *Anopheles funestus* and *A. arabiensis* to penetrate LLINs and its effect on their mortality

**DOI:** 10.12688/wellcomeopenres.18242.2

**Published:** 2023-02-06

**Authors:** Felician Clement Meza, Letus L Muyaga, Alex Julius Limwagu, Dickson Wilson Lwetoijera

**Affiliations:** 1 Environmental Health and Ecological Sciences, Ifakara Health Institute, DAR ES SALAAM, N/A, 14112, Tanzania; 2 Life Science and Bioengineering, Nelson Mandela Africa Institution of Science and Technology, Arusha, Tanzania

**Keywords:** Long -lasting insecticidal nets, Malaria vectors, Anopheles funestus, Anopheles arabiensis, Malaria, Tanzania

## Abstract

**Background:** Variation in mosquito body size and the ability to penetrate long-lasting insecticide-treated nets (LLINs) remains unknown. This study evaluated the ability of *Anopheles funestus* and *A. arabiensis* to penetrate commercially available treated and untreated bednets and how this behaviour affects mosquito mortality.

**Methods:** Three types of LLINs; DawaPlus 2.0, PermaNet 2.0, Olyset 2.0, and untreated (Safi Net) were tested inside a semi-field system. One hundred 3–5-day-old female *A. funestus* and *A. arabiensis* were released in a chamber with a sleeping adult volunteer under a treated or untreated bednet. Mosquitoes that penetrated inside the nets were collected every two hours using a mouth aspirator. Live mosquitoes were put in paper cups, fed on glucose *ad libitum* and their mortality rate was monitored for 48 h.

**Results:** The ability of *A. funestus* to penetrate treated and untreated bednets was significantly higher than for *A. arabiensis* for all three LLIN net types ( *P*<0.001). For both species the penetration rate was higher for untreated bednets than treated ones except for the Olyset net. Regardless of the assessed mosquito species, all the mosquitoes that penetrated the net, successfully blood-fed on the sleeping volunteer. Compared to *A. arabiensis*, significant mortality was recorded for *A. funestus* that were caught inside Olyset nets within 48 hrs of monitoring ( *P*<0.001).

Conclusions:

These findings demonstrate the ability of *A. funestus* and *A. arabiensis* mosquitoes to penetrate the human-occupied treated and untreated bednets. Despite this ability, mosquitoes that penetrated the bednet succumbed to death within two days.

## Introduction

Amongst vector control strategies to combat malaria, the use of long-lasting insecticide-treated nets (LLINs) has been key in sub-Saharan Africa, which bears the brunt of the disease and accounts for nearly 95% of all associated cases and deaths. In the last two decades, LLINs contributed to nearly 70% of the progress made ^1^. In addition, a recent study demonstrated the contribution of LLINs in the reduction of disease incidence and malaria-related child mortality from all causes by 17% compared to no nets ^2^. LLINs not only provide a physical barrier against mosquitoes that attempt to bite its sleeping occupant, it also diverges mosquitoes attempting to enter the house, kills mosquitoes that make physical contact with the mesh, and reduces mosquito survival ^3^.

Similar to other African countries, Tanzania relies heavily on LLINs as the standard of care against malaria transmission. The use of such effective tools in Tanzania goes back 20 years, a period during which its access, coverage and proper usage was maintained and implemented through under-five catch-up campaign ^4^, universal coverage campaign, and keep-up campaign, that encompass a piloted school net programmes ^5^, with special emphasis on vulnerable populations ^6^. These initiatives resulted in significant reductions of malaria, exemplified by the recent empirical evidence of near-elimination of the historically dominant malaria vector *Anopheles gambiae* in Kilombero valley ^7^; a similar trend has been documented in other part of East Africa ^8^. More recently, in the Kilombero Valley and in other parts of Africa, there is growing evidence that on-going residual malaria transmission is mediated by *A. funestus* and *A. arabiensis* ^9^. While the latter species represents the most abundant malaria vector, the former predominates most of the transmission ^9, 10^.

The constant use of LLINs and continuous interaction of LLINs and targeted malaria vectors have brought major threats such as resistance to insecticides that are used for impregnation ^11^ as well as emergence of behavioural avoidance strategies of malaria vectors ^10, 12^. For instance, *A. arabiensis* has adapted to avoid contacting treated nets by biting and resting outdoors, feeding on cattle instead of human ^8, 10, 12^. *Anopheles funestus*, on the other hand, is highly anthropophilic ^10^, has high vectorial competence for *Plasmodium* spp., is highly resistant to pyrethroids throughout Africa and has adapted biting times that avoid interventions such as LLINs and maintains malaria transmission during the drier parts of the year ^9^. Previous studies have documented the resistance ^13, 14^ and behavioural avoidance ^12, 15^ of *A. arabiensis* and *A. funestus* of the insecticides used in LLINs. However, the possibility of these major malaria vectors to evade and penetrate the mesh of LLINs and reach and feed on their designated host is yet to be established.

LLIN effectiveness against malaria vectors depends on the physical integrity (holes), insecticide content, bio-efficacy, use, and attrition over time ^16^. However, the mesh size of the net fabric that prevents mosquitoes from penetrating it may have an important contribution to the overall protection offered by the net. Many studies have focused on monitoring of the physical integrity ^17, 18^, insecticide activity, bio-efficacy, usage and net loss ^19^ as recommended by the World Health Organization (WHO) guidelines ^20^, leaving out the interaction between the mesh size of the net and malaria vector behaviour. A few studies have described net penetration and the interaction of mosquitoes with different simulated net hole sizes ^21^, or the wide mesh sizes of the net for *Culex* spp. mosquitoes ^22^. For nets to be effective in preventing mosquitoes from entrance or penetration, WHO recommend that the mesh size of the net fabric should be 156 or 196 holes per square inch or between 1.2–1.5 mm ^23^. Nevertheless, the mosquitoes with small body sizes and their ability to penetrate the mesh of commercially available LLINs remains unknown. We hypothesized that mosquitoes with a small body size, such as *Anopheles funestus,* and possibly the generally larger *A. arabiensis* can penetrate commercially available bednets (1.2–1.5 mm mesh treated or untreated) and access and feed on the host before they die, which might be one of the causes for residual malaria transmission. This study aimed to evaluate the ability of *A. funestus* and *A. arabiensis* to pass through the mesh of commercially available treated or untreated bed nets and how passage impacts their survival post penetration.

## Methods

### Study area and site

Experiments were carried out inside a semi-field system (SFS) of the Ifakara Health Institute located at Kining’ina village (8.11417 S, 36.67484 E), Kilombero Valley, southern Tanzania. The Kilombero Valley has seen a notable scale-up of LLINs over the past 20 years, that led to undetectable levels of the major malaria vector *A. gambiae* ^7^. Despite this success, most malaria transmission within this area today is mediated by *A. funestus*, a species that feeds almost exclusively on humans (anthropophilic) and rests indoor after feeding (endophilic) and is strongly resistant to the commonly used pyrethroid insecticides, both metabolically and physiologically ^9, 24^. 

### Adult collection and establishment of *A. funestus* colony

Field collection of adult *A. funestus* to establish a first generation (F1) in the semi-field insectary was undertaken between January and June 2021. Adult *A. funestus* were sampled and collected from 10 houses selected randomly in Ikwambi (7.98033°S, 36.81701°E) and Sululu (8.00324°S, 36.83118°E) villages, using CDC light traps and Prokopack aspirators. One CDC light trap, modified with a long collection bag or cups to prevent trapped mosquitoes from drying out because of the CDC fan, was used to sample *A. funestus* in each household. The traps were positioned near the bednet’s occupant in the evening and retrieved in the morning for mosquito collection. Sampling of indoor resting mosquitoes using the Prokopack aspirator was done every day in the morning starting at 6 a.m. and finishing around 7 a.m. Mosquitoes were morphologically identified to species level in the field using the Gillies and Coetzee identification key ^25, 26^. Afterwards, *A. funestus* were kept in a small cage with 10% glucose to feed on and transported to the insectary in the semi-field at Kining’ina. Upon arrival, mosquitoes were blood-fed on a chicken and allowed to lay eggs individually in a paper cup for the establishment of an *A. funestus* colony.

In the insectary, larvae resulting from laid eggs, were held in rearing basins of 32 cm diameter and 5-L holding capacity and fed on ground fish food, Tetramin®, until they developed into pupae ^27^. Pupae were collected every day and transferred to 0.5-L plastic cups placed inside the 0.3×0.3×0.3 m cage covered with netting prior to adult emergence. Emerging adults were morphologically identified as *A. funestus* using identification keys by Gilles and Coetzee ^28^. A subsample of 100 *A. funestus* was confirmed to species level using PCR ^29^. *A. arabiensis* were obtained from the Kining’ina insectary colony in which larvae are fed on ground fish, Tetramin® (Tetra, Melle, Germany), and adults are maintained in the same standard rearing conditions of 12 h:12 h photoperiod, 25–27 °C ambient temperature and (70–85%) relative humidity as *A. funestus* colony.

### Semi-field system experiments

**Figure 1.**
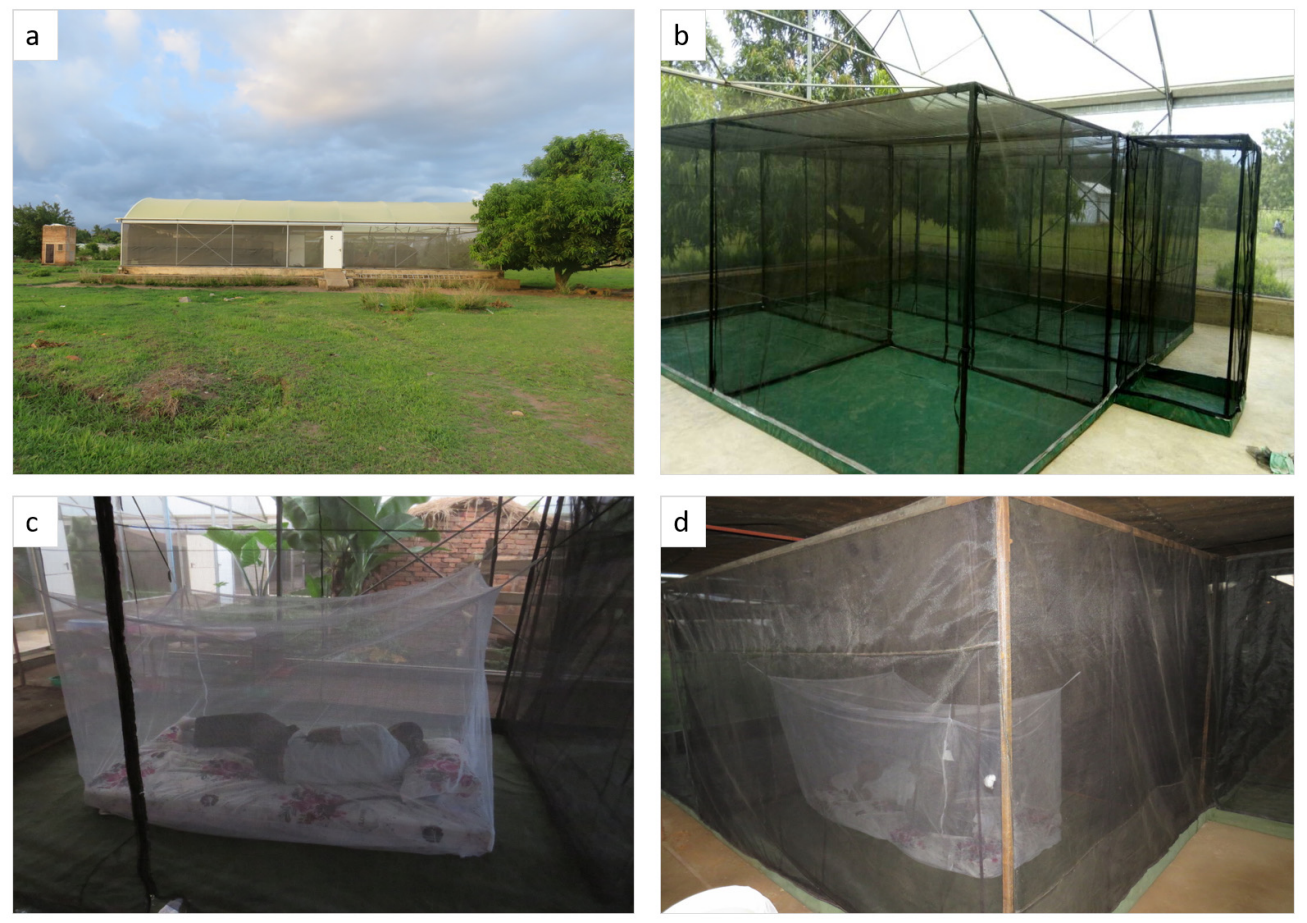
( **a**) Semi field system in Kining`ina, ( **b**) a 3×3×2 m cages that were constructed inside one of the chambers of the Semi-field system, ( **c**) A sleeping volunteer inside the constructed cage, sleeping under bed net and ( **d**) Orientation view of a sleeping volunteer in a cage.

The experiment was conducted inside two chambers of the four chambers-cage constructed inside the SFS measuring 3×3×2 m using a double layer of fiber glass nets on the outside, and lined with small-mesh fabric in the inside to prevent mosquito escape ( Figure 1a, b and c). Of the two chambers, one was used treated net and the other for untreated net set ups ( Figure 1d). Three types of insecticide-treated nets were tested, namely DawaPlus 2.0 (Tana Netting FZ LLC, Dubai), PermaNet 2.0 (Vestergaard S.A, Switzerland), Olyset 2.0 (Sumitomo Chemical Co., Ltd, Japan), besides an untreated SafiNet (A to Z Textile Mills Limited Tanzania). DawaPlus® 2.0 and PermaNet 2.0 consist of 100% polyester, with deltamethrin coated on 100 deniers at 80 mg/m ^2^ and 55 mg/m ^2^, respectively. Both nets have a mesh size of 160 per square inch ^30^. OlysetNet® is made of 100% polyethylene, with permethrin incorporated in 150 deniers at 50 mg/m ^2^ with a mesh size of 160 holes per square inch, which is characteristically large (4×4 mm) ^30^. The SafiNet that was used has the control, of 100% polyester, and has 75 deniers and mesh size of 156 holes per square inch.

Each test chamber was fitted with a mattress on the floor to make a bed for a sleeping adult volunteer protected with a treated or untreated net in the treatment or control chambers, respectively ( Figure 1c and d). Experiments started at 6.00 pm by releasing 100 female *A. funestus* or *A. arabiensis* in the presence of a volunteer. In both chambers, all mosquitoes were to be released in less than one minute. Every two hours the volunteer woke up to collect mosquitoes that had penetrated the net using a mouth aspirator and put these in a labelled paper cup. In the morning, all dead and live mosquitoes were collected from inside and outside the net. Live mosquitoes from inside and outside the net were put in paper cups, fed *ad libitum* on 10% glucose solution, and monitored for mortality for 48-hours consecutive on 24-hours cycle and delayed mortality ^31^. Six replicates were performed for each net type.

### Statistical analysis

A generalized linear mixed effects models (GLMM) following binomial distribution with a logit function built on the *lme4* package ^32^, was used to determine whether *A. funestus* and *A. arabiensis* successfully penetrated the treated or untreated bednets in the semi-field. The number of *A. funestus* or *A. arabiensis* caught inside the net denoted as success, and those caught outside the nets denoted as failure were modeled as a response variable, and replicates as a random variable. The net status (treated or untreated net), species ( *A. funestus* or *A. arabiensis*) and collection site (inside the net or outside nets) were modeled as fixed variables. Model coefficients were exponentiated to obtain the odds ratio, with their respective *P-*values at 95% confidence intervals (CIs).

## Results

**Figure 2.**
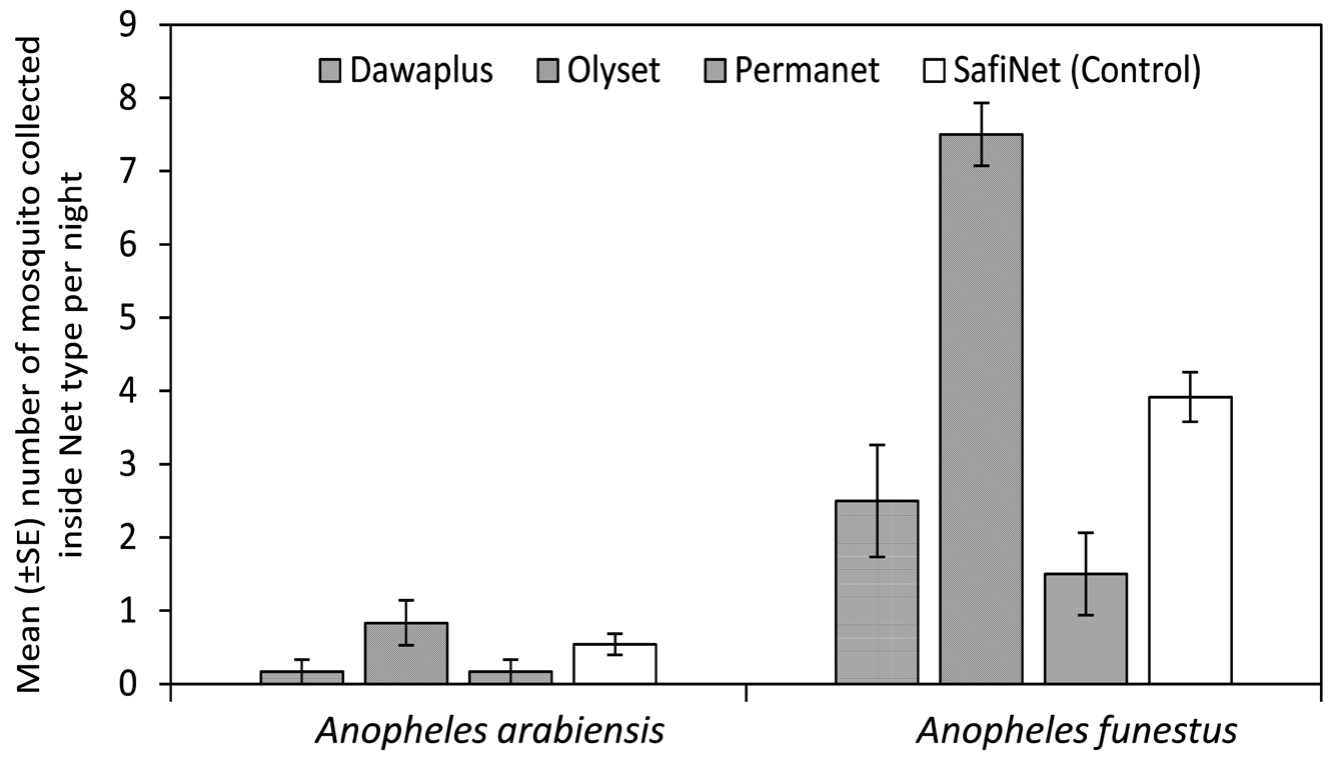
Mean number of *Anopheles arabiensis* and *Anopheles funestus* collected inside different net types.

Overall, the ability of *A. funestus* to penetrate treated and untreated netting was higher than that of *A. arabiensis* for all four net types ( Figure 2, Table 1). Both species more easily penetrated untreated than treated nets with the exception of the Olyset net, which was penetrated by significantly higher number of mosquitoes compared to the untreated net ( *p*<0.001) ( Figure 2, Table 1). The mean number (±S.E) of *A. arabiensis* was less than one per night across all net types, whereas for *A. funestus* this was up (7.5±0.31) and (3.9±0.34) mosquitoes in a treated and untreated nets respectively. Regardless of the assessed mosquito species, almost all mosquitoes that penetrated the net, successfully blood-fed on the sleeping volunteer ( Table 2). Moreover, *A. funestus* showed significantly higher ability of penetrating DawaPlus, Olyset, and PermaNet nets (OR 0.02 [0.016, 0.288], *p* <0.001), (OR 0.03 [0.027, 0.042], *p*<0.001), and (OR 0.016 [0.011, 0.022], *p* <0.001) respectively, when compared to *A. arabiensis* ( Table 1). The 48-h mean mortality was similar for both species that penetrated treated DawaPlus 2.0 and PermaNet 2.0 nets ( Table 3). In comparison to *A. arabiensis*, significant mortality was recorded for *A. funestus* that were caught inside Olyset Nets within 48 hrs of monitoring ( *p* <0.001).

**Table 1.** Penetration rate of *A. funestus* caught outside the net with the model output showing the ability of mosquitoes to penetrates (inside the net) in test chambers.

Net type	Reference item [1*]	Odd ratio	95%CI	P value
**DawaPlus Net 2.0**	Safi Net	NA	NA	NA
***A. funestus***	*A. arabiensis*	1.03	[0.68, 1.48]	0.580
**Collected (Inside a net)**	Outside a net	0.02	[0.02, 0.29]	<0.001
**Olyset Net 2.0**	Safi Net	NA	NA	NA
***A. funestus***	*A. arabiensis*	1.04	[0.93, 1.17]	0.450
**Collected (Inside a net)**	Outside a net	0.03	[0.03, 0.04]	<0.001
**PermaNet 2.0**	Safi Net	NA	NA	NA
***A. funestus***	*A. arabiensis*	1.02	[0.92, 1.15]	0.669
**Collected (Inside a net)**	Outside a net	0.016	[0.01, 0.02]	<0.001

CI = confidence interval, NA = not applicable because it’s a reference with value [1*]

**Table 2.** Mean (±SE) number of blood fed mosquitoes that were caught inside treated and untreated nets.

Species	DawaPlus Net		Olyset Net		PermaNet		SafiNet	
Mean (±S.E)	Fed	Unfed	Fed	Unfed	Fed	Unfed	Fed	Unfed
***A. arabiensis***	0.17 ± 0.17	0	0.83 ± 0.31	0	0.17 ± 0.16	0	0.50 ± 0.13	0.04 ± 0.04
***A. funestus***	2.50 ± 0.76	0	6.67 ± 0.31	0.83 ± 0.54	1.50 ± 0.56	0	3.88 ± 0.34	0.04 ± 0.04

**Table 3.** 48-hours mortality of mosquitoes penetrated the treated and untreated nets. Model output to show the effects of net status (Treated or Untreated) on the survival of *A. funestus* and *A. arabiensis* in the test chambers.

Net type	Reference item [1*]	Odd ratio (at 48-hours)	95%CI	P value
**DawaPlus 2.0 **				
**Net status (Treated) **	Safi Net (Untreated)	12.18	[8.23, 18.1]	<0.001
***A. funestus***	*A. arabiensis*	1.02	[0.79, 1.31]	0.897
**Olyset 2.0**				
**Net status (Treated)**	Safi Net (Untreated)	12.20	[8.26, 17.9]	<0.001
***A. funestus***	*A. arabiensis*	1.89	[1.46, 2.45]	<0.001
**PermaNet 2.0**				
**Net status (Treated) **	Safi Net (Untreated)	7.78	[5.56, 10.9]	<0.001
***A. funestus***	*A. arabiensis*	1.12	[0.87, 1.44]	0.373

CI = confidence interval

## Discussion

This study documented the ability of *A. funestus* and *A. arabiensis* mosquitoes to penetrate commercially available treated and untreated nets. Worryingly, *A. funestus* showed a marked ability of penetrating all nets types compared to *A. arabiensis.* Among other factors ^9, 12^ this ability of *A. funestus* to penetrate intact nets, might be an additional risk for sustained malaria transmission across Africa ^9, 11^. This phenomenon signals an alarming situation for malaria mosquitoes control efforts that heavily relies on the use of LLINs, which its ability to prevent mosquito bites that exceeds 80% come from physical barrier and integrity ^11, 33^. Although the numbers of both mosquito species that penetrated the nets was relatively low, the fact that the majority that did successfully blood fed on the volunteers, its effect on malaria transmission is likely to be noticeable if these numbers are magnified across mosquito populations under real life settings.

Although wing length for both mosquito species were not measured in the current study, estimates of mosquito body sizes of F1-laboratory reared mosquitoes were extracted from recent studies that used the same colonies and/or collected adults for F1 from the same village ^34, 35^. Compared to *A. arabiensis* with relatively large body size (2.6 – 3.6 mm), the marked ability of *A. funestus* to penetrate all net types might be attributed to its small body size (2.6 – 2.7 mm) relative to the mesh holes size of the net ^7, 35^. Olyset mesh size of 4 mm x 4 mm that ensure better ventilation to the bednet occupant, might also be the reason for the recorded significantly high number of mosquitoes penetrating this net type compared to other nets. Under field settings, the penetration and successful feeding by wild and resistant *A. funestus* population on bednet occupant(s), especially in a situation where nets are not treated and/or its fabric integrity is compromised, might accelerate and exacerbate malaria transmission in communities ^36^.

Treated nets resulted high mortality in *A. arabiensis* and *A. funestus,* which highlights the importance of incorporated insecticides in LLINs over untreated nets. For both species, only a small proportion that were collected outside the net died, probably from short-contact with a treated net or from insecticide vapours ^37, 38^. Almost all *A. arabiensis* that penetrated treated nets, although in low numbers, died within 48 h of monitoring. This might be due to full susceptibility of the used mosquitoes from the laboratory to the insecticides in the nets. On the other hand, *A. funestus* that penetrated the Olyset net and successful fed died within 48 h of monitoring. Although previous reports have confirmed resistance of *A. funestus* to permethrin, a pyrethroid used in the LLINs used in this study ^9, 24^, the observed increase in mortality might have been due to prolonged exposure to insecticide as mosquito that were trapped inside the net. Majority of *A. funestus and A. arabiensis* that penetrated the net were blood-fed, and died within 48 h of monitoring. The recorded delayed mortality ( *i.e*. dying within 48 h and not 24 h) might have been due to increased insecticide tolerance as the results acquired blood meals ^39^.

While debate on the impact of insecticide resistance in reducing protective effectiveness of LLINs has remained inconclusive ^40, 41^ findings from this study highlights the benefits of incorporated insecticides in causing mortality even to resistant mosquitoes, such as *A. funestus*, that penetrated the nets. These findings point out the importance of considering future modification of mesh sizes that offer a complete bite protection by preventing mosquitoes to penetrate the net without compromising ventilation. Because only new nets were tested in the current study, further studies might be warranted to assess the penetration rate and associated consequences on their fitness as the nets get old. In addition, these findings can be coupled with community perception on mosquito penetration to different nets in question.

Nevertheless, the current study had number of limitations. First, no direct wing length measurement that were taken for mosquitoes used in this study, instead estimate of mosquito body sizes were drawn from recent studies that measured the body of the same mosquito colonies used. Second, no testing of insecticide resistance that was carried out to confirm the resistance within *A. funestus.* The claimed resistance within *A. funestus* was based on the previous studies conducted in the similar location ^13^. Third, no attempts were made to establish if the recorded penetration was a mosquito learned behaviour to maximize its feeding success. A recent study documented learning behaviour in *Aedes* and *Culex* mosquitoes that enables them avoid lethal contact via detection of insecticide smell ^42^. Fourth, the landing and penetration activities of *A. funestus* on the nets were not recorded. Of importance, this can be achieved by setting up camera traps that are already used in mosquito behaviour and ecological studies ^43^. This will provide information on which part of the net do mosquitoes prefer to penetrate most, with potential to guide the customization of mesh size and insecticide impregnation at specific areas of the net.

## Conclusion

*A. funestus and A. arabiensis* have the ability to penetrate treated and untreated bednets and contact their preferred host due to their small size relative to net mesh hole sizes. This study highlights the limitation of current net-mesh size in offering biting proof against *A. funestus* and *A. arabiensis,* the dominant malaria vector, and counterbalance importance of impregnated insecticides on mosquito mortality. Owing to its functional value on the overall bednet protective effect, future studies on the WHO recommended new generation bednets should also consider assessing its penetration by targeted mosquitoes species and effect on its fitness.

## Ethical approval

The Institutional Review Board of the Ifakara Health Institute (IHI/IRB/EXT/No: 24-2021) and the Medical Research Coordination Committee of the National Institute for Medical Research in Tanzania (NIMR/HQ/R.8a/Vol.IX/3477) approved the study.

## Data Availability

Figshare: Underlying data for “ **The ability of *Anopheles funestus* and *A. arabiensis* to penetrate LLINs and its effect on their mortality**” https://doi.org/10.6084/m9.figshare.20621664 ^44^ This project contains the following underlying data: Data file 1 (Mosquito penetrating different bed nets types and its feeding success and mortality). Data are available under the terms of the Creative Commons Zero "No rights reserved" data waiver (CC0 1.0 Public domain dedication).
