# Supplementary material for: The ability of Anopheles funestus and A. arabiensis to penetrate LLINs and its effect on their mortality
Source: Wellcome Open Res. 2023 Apr 6;7:265. Originally published 2022 Oct 18. [Version 3] doi: 10.12688/wellcomeopenres.18242.3 (PMC10039320; doi:10.12688/wellcomeopenres.18242.3)
Supplement: Peer review history

# Peer review: Reviewer response for version 2

**DOI:** 10.21956/wellcomeopenres.21064.r55708

**Published:** 2023-03-24

**Authors:** Wolfgang Richard Mukabana

**Affiliations:** 1 School of Biological Sciences, University of Nairobi, Nairobi, Kenya

The authors of this study have provided data for what we would assume has already been published by others. The idea was to find out whether two species of malaria mosquitoes present in Africa, namely *An. funestus* and *An. arabiensis*, are able to penetrate commonly used brands of insecticide treated bed nets (ITNs) and bite occupants. The authors found out that the mosquitoes penetrated the ITNs and bit occupants. These findings are unexpected despite the shortcomings of the study (insecticide resistance in the mosquitoes was not measured, mosquito sizes were not measured, no attempts were made to find out if net penetration was a mosquito learnt behavior and actual mosquito penetration events were not recorded), which the authors discussed. There it at least one more information segment that is missing i.e. it is not known if released mosquitoes were pre-starved, and if yes, for how long. Hunger is an important driver for host seeking and may have triggered the mosquitoes to 'work harder' to reach their blood meal hosts. This issue should be clarified and deliberated upon in the discussion section. Furthermore, a graph showing mortality versus time of collection by the ITN occupant can help to further explain the findings. Lastly, the fill colors of the bars for Dawaplus, Olyset and Permanet nets in figure 2 should be properly differentiated to improve clarity and understanding of the data.

Is the work clearly and accurately presented and does it cite the current literature?

Yes

If applicable, is the statistical analysis and its interpretation appropriate?

Yes

Are all the source data underlying the results available to ensure full reproducibility?

Yes

Is the study design appropriate and is the work technically sound?

Yes

Are the conclusions drawn adequately supported by the results?

Yes

Are sufficient details of methods and analysis provided to allow replication by others?

Yes

Reviewer Expertise:

Public Health Entomology with a great focus on malaria.

I confirm that I have read this submission and believe that I have an appropriate level of expertise to confirm that it is of an acceptable scientific standard.

# Peer review: Reviewer response for version 1

**DOI:** 10.21956/wellcomeopenres.20221.r53001

**Published:** 2022-11-23

**Authors:** Alphaxard Manjurano

**Affiliations:** 1 Department of parasitology, Mwanza Medical Research Centre, National Institute of Medical Research in Tanzania, Mwanza, Tanzania

There are a few primary concerns identified in the manuscript, as stated below.

 Specific comments:

**Methods:**

1. Sampling of indoor resting mosquitoes using the Prokopack aspirator was done every day in the morning. There is a need to expand this statement to state the exact timing of the sampling as morning is the period from sunrise to noon. It is that the mosquitoes are likely to escape when it becomes day time. Therefore, timing should be precisely mentioned.
2. For the data analysis section, more details need to be included here overall. More detail should be included for this, and its justification for use of generalized linear mixed model to evaluate the mean numbers indicated in the results. The appropriate test for mean calculation should be included.

**Results:**

1. Figure 2 which shows the Mean number of *Anopheles arabiensis *and *Anopheles funestus *collected inside different net types is difficult to follow except the control bar, the shading of the different nets has similar patterns. I suggest this to be revised for easy follow-up to the readers of the manuscript.
2. Table 1 is difficult to interpret as it is not clear which is the reference (type of mosquitoes or location of the net?). This also applies to table 2.
3. The statement “Of all mosquitoes that penetrated the net, regardless of the species, >90% successfully blood-fed on the sleeping volunteer (Table 2)’’ is not supported by the results indicated in the table as the table indicates Mean number of blood fed mosquitoes that were caught inside treated and untreated nets, and not percentage

**Discussion:**

1. The statement “Although the absolute numbers of both mosquito species that penetrated the nets was relatively low”. There is a difference between absolute numbers and mean numbers. Therefore, there is no evidence showing numbers of mosquitoes.

Is the work clearly and accurately presented and does it cite the current literature?

Yes

If applicable, is the statistical analysis and its interpretation appropriate?

I cannot comment. A qualified statistician is required.

Are all the source data underlying the results available to ensure full reproducibility?

No source data required

Is the study design appropriate and is the work technically sound?

Yes

Are the conclusions drawn adequately supported by the results?

Yes

Are sufficient details of methods and analysis provided to allow replication by others?

Yes

Reviewer Expertise:

molecular biology, entomology, immunology and genetic epidemiology

I confirm that I have read this submission and believe that I have an appropriate level of expertise to confirm that it is of an acceptable scientific standard.
